# Repetition-to-Repetition Differences Using Cluster and Accentuated Eccentric Loading in the Back Squat

**DOI:** 10.3390/sports6030059

**Published:** 2018-07-08

**Authors:** John P. Wagle, Christopher B. Taber, Kevin M. Carroll, Aaron J. Cunanan, Matt L. Sams, Alexander Wetmore, Garett E. Bingham, Brad H. DeWeese, Kimitake Sato, Charles A. Stuart, Michael H. Stone

**Affiliations:** 1 Center of Excellence for Sport Science and Coach Education, Department of Sport, Exercise, Recreation, and Kinesiology, East Tennessee State University, Johnson City, TN 37614, USA; carrollk@etsu.edu (K.M.C.); cunanan@etsu.edu (A.J.C.); wetmore@etsu.edu (A.W.); binghamg@etsu.edu (G.E.B.); deweese@etsu.edu (B.H.D.); satok1@etsu.edu (K.S.); stonem@etsu.edu (M.H.S.); 2 Department of Physical Therapy and Human Movement Science, Sacred Heart University, Fairfield, CT 06825, USA; taberc@sacredheart.edu; 3 Department of Exercise Science and Health Education, LaGrange College, LaGrange, GA 30240, USA; matt.l.sams@gmail.com; 4 Department of Internal Medicine, Quillen College of Medicine, East Tennessee State University, Johnson City, TN 37604, USA; stuartc@etsu.edu

**Keywords:** resistance training, eccentric overload, programming, potentiation, rate of force development, power, strength

## Abstract

The current investigation was an examination of the repetition-to-repetition magnitudes and changes in kinetic and kinematic characteristics of the back squat using accentuated eccentric loading (AEL) and cluster sets. Trained male subjects (age = 26.1 ± 4.1 years, height = 183.5 ± 4.3 cm, body mass = 92.5 ± 10.5 kg, back squat to body mass ratio = 1.8 ± 0.3) completed four load condition sessions, each consisting of three sets of five repetitions of either traditionally loaded straight sets (TL), traditionally loaded cluster sets (TLC), AEL cluster sets (AEC), and AEL straight sets where only the initial repetition had eccentric overload (AEL1). Eccentric overload was applied using weight releasers, creating a total eccentric load equivalent to 105% of concentric one repetition maximum (1RM). Concentric load was 80% 1RM for all load conditions. Using straight sets (TL and AEL1) tended to decrease peak power (PP) (*d* = −1.90 to −0.76), concentric rate of force development (RFD_CON_) (*d* = −1.59 to −0.27), and average velocity (MV) (*d* = −3.91 to −1.29), with moderate decreases in MV using cluster sets (*d* = −0.81 to −0.62). Greater magnitude eccentric rate of force development (RFD_ECC_) was observed using AEC at repetition three (R3) and five (R5) compared to all load conditions (*d* = 0.21–0.65). Large within-condition changes in RFD_ECC_ from repetition one to repetition three (∆REP_1–3_) were present using AEL1 (*d* = 1.51), demonstrating that RFD_ECC_ remained elevated for at least three repetitions despite overload only present on the initial repetition. Overall, cluster sets appear to permit higher magnitude and improved maintenance of concentric outputs throughout a set. Eccentric overload with the loading protocol used in the current study does not appear to potentiate concentric output regardless of set configuration but may cause greater RFD_ECC_ compared to traditional loading.

## 1. Introduction

Strength-power adaptations to resistance training are primarily determined by the mode of exercise which is implemented and type of loading encountered [1]. The development of strength and power can be optimized through proper management of acute training variables such as sets, reps, rest periods, and exercise order [2]. However, greater degrees of variation and novelty of stimulus are required to continue to drive changes in athletes with an advanced training status [3,4]. Novelty and variation must be systematically planned, sequenced, and with consideration of the multi-faceted nature of the demands of sporting actions. Therefore, coaches must make creative manipulations of the more nuanced variables to properly disrupt homeostasis with two of the most prevalent being accentuated eccentric loading (AEL) and inter-repetition rest. 

Accentuated eccentric loading is an advanced training tactic aiming to exploit the muscle’s ability to produce greater force during eccentric muscle actions compared to isometric and concentric actions [5,6]. This method is prescribed for movements that require coupled eccentric–concentric actions (e.g. back squat, bench press), using eccentric loads in excess of the concentric prescription. Ideally, this is achieved while imparting minimal interruption to the natural mechanics of the chosen exercise [7]. Accentuated eccentric loading has been explored in several studies using both upper [8,9,10,11] and lower body [10,11,12] exercises. AEL has demonstrated positive effects on concentric performance compared to traditional loading patterns [8,12] though not all studies agree [9,10,11]. The inconsistent nature of the existing evidence may be largely due to the discrepancy in both eccentric and concentric loading, means of application, and exercise selection among other confounders. Furthermore, as AEL typically requires time between repetitions to reload the eccentric load, it is possible the inter-repetition rest may explain some of the purported benefits of AEL [13]. 

Inter-repetition rest typically termed a cluster set is an efficacious programming tactic independent from its potential influence on AEL. Previous literature has demonstrated that various cluster set arrangements can offset the loss in movement velocity and maintain power outputs [14,15,16]. Interestingly, the potentiating effects of cluster sets appear to be more substantial when prescribed to athletes with an advanced training age [17], suggesting clusters may be more appropriately applied as an advanced tactic [18]. Some have suggested this may be the case regarding AEL as well [7], though such a hypothesis must be explored further. To exploit the potential advantages of the aforementioned strategies, an intimate knowledge of their acute characteristics is valuable in hypothesizing the chronic response.

Though previous literature has recently elucidated foundational kinetic and kinematic characteristics of AEL and cluster sets [13], repetition-to-repetition magnitudes and maintenance have not yet been examined. Therefore, the purpose of the current investigation was to build upon previous findings [13] and explore the repetition-to-repetition kinetic and kinematic differences between potential programming tactics in the back squat. Specifically, the authors aimed to determine the effects of (1) eccentric overload and (2) inter-repetition rest on the magnitude and repetition-to-repetition changes of rate-related eccentric and concentric characteristics. The findings of the current investigation aim to inform resistance training programming decisions by providing more robust information regarding the separate and combined effects of these increasingly prevalent training strategies. 

## 2. Materials and Methods

### 2.1. Subjects

Eleven resistance-trained males (age = 26.1 ± 4.1 years, height = 183.5 ± 4.3 cm, body mass = 92.5 ± 10.5 kg, back squat to body mass ratio = 1.8 ± 0.3) volunteered for the current investigation. To qualify, subjects were required to have spent at least the past year in a weekly resistance training program that consistently included back squats. Urinary specific gravity was determined prior to any data collection using a refractometer (Atago, Tokyo, Japan) to ensure the subjects’ hydration status would not influence the results [19]. All subjects read and signed a written informed consent and the procedures were approved by the university’s Institutional Review Board. 

### 2.2. Procedures

Dynamic strength was measured using a previously established one-repetition maximum back squat (1RM) protocol [20]. The 1RM was achieved by each subject within three maximal attempts and was preceded by a standardized squat warm-up based on each subject’s self-reported 1RM back squat. The final successful 1RM attempt was subsequently used in determining load prescription for experimental loading conditions.

The initial experimental back squat session began a minimum of 48-hours following each subject’s dynamic strength testing. Experimental sessions were assigned in a random order using an online randomization tool [21]. Following the initial load condition, each subsequent session was separated by seven days and executed at the same time of day for each subject. Between sessions, subjects were permitted to train typical to their respective routines, except for complete rest 48 hours prior to any data collection. The general and specific warm-up was identical to that used in dynamic strength testing [20], with loading adjusted based on the tested 1RM. Subjects performed three sets of five repetitions of the barbell back squat for each prescribed condition, with each set separated by three minutes of passive rest. Concentric intensity for all load conditions was 80% 1RM [22]. Accentuated eccentric loading totaled 105% of 1RM [8,22,23] and was applied to prescribed repetitions via weight releasers (Monster Grips, Columbus, OH, USA) [12,23,24]. Subjects were strongly verbally encouraged in the same manner during each session to perform the concentric phase of the squat as explosively as possible. 

Four loading conditions which were typical of athletic populations were used to better understand the uniqueness of different programming strategies. Traditionally loaded “straight sets” (TL) were completed with no intra-set rest, completing each of the five back squat repetitions per set consecutively. No more than three seconds were allowed between repetitions. Two load conditions allowed intra-set rest, which is the basis for a cluster set [18]. Traditionally loaded cluster sets (TLC) were completed with identical load to TL, but 30 seconds of intra-set standing rest was prescribed where the subjects placed the barbell on the safety hooks of the squat rack between repetitions. During the accentuated eccentric load cluster set condition (AEC), all five repetitions of the back squat were completed with eccentric overload (105% 1RM) with otherwise identical procedures to those of TLC. The accentuated eccentric load “straight set” condition (AEL1) added an eccentric overload to the first repetition of each set only and subsequent repetitions were completed using procedures identical to TL. The AEL1 condition aimed to examine the effects of AEL without intra-set rest.

Data were collected using a dual force plate design (2 × 91 cm × 45.5 cm force plates, Roughdeck HP, Rice Lake, WI, USA) inside a custom-built apparatus with data sampled at 1,000 Hz [13]. Four linear position transducers (PT101-0100-H14-1120, Celesco, Chatsworth, CA, USA) were attached to the top of the custom-built apparatus and recoil wires were attached to the each of the ends of the barbell just inside where the plates were loaded [13]. The linear position transducers were synchronized with the force plates using a custom LabVIEW (version 7.1, National Instruments, Austin, TX, USA) program. Data were processed using RStudio (Version 1.0.153, RStudio, Inc., Boston, MA, USA). To account for and diminish noise, a digital Butterworth 2nd order low-pass filter was applied. Eccentric and concentric phases were confirmed by the displacement values obtained from the linear position transducers. Repetition-to-repetition values and changes in peak power (PP), eccentric rate of force development (RFD_ECC_), concentric rate of force development (RFD_CON_), and concentric average velocity (MV) were assessed for each load condition. The slope between eccentric peak force and the force value 250 ms prior to eccentric peak force was used to determine RFD_ECC_ [25]. The timepoint of 250 ms was chosen to reflect the upper limit of time in which stored eccentric energy may be used to enhance the subsequent concentric action rather than dissipated as heat [26]. Concentric rate of force development was determined using the concentric peak force and the force value 250 ms prior [27]. 

### 2.3. Statistical Analyses

Descriptive statistics for each load condition including mean and 90% confidence interval (CI) were calculated using all three sets for the first (R1), third (R3), and fifth (R5) repetitions as well as the change from R1 to R3 (∆REP_1–3_) and change from R1 to R5 (∆REP_1–5_) (Table 1, Table 2, Table 3 and Table 4). Within subject reliability for each dependent variable was assessed using coefficient of variation (CV) and intraclass correlation coefficients (ICC (2,1)), with every repetition performed being considered in determining reliability [28,29]. Coefficient of variation was calculated using the mean and standard deviation of each dependent variable. Within-condition Cohen’s *d* effect sizes (ES) and 90% CI were calculated for ∆REP_1–3_ and ∆REP_1–5_ using the average of each individual’s effect statistic [30]. Between-condition Cohen’s *d* ES and 90% CI were calculated for each dependent variable [30]. Effect sizes were interpreted with magnitude thresholds of 0–0.2, 0.2–0.6, 0.6–1.2, 1.2–2.0, and 2.0 and above as trivial, small, moderate, large, and very large [31]. Statistical analyses were performed using Microsoft Excel^TM^ (Version 1806, Redmond, WA, USA).

## 3. Results

**Figure 1:**
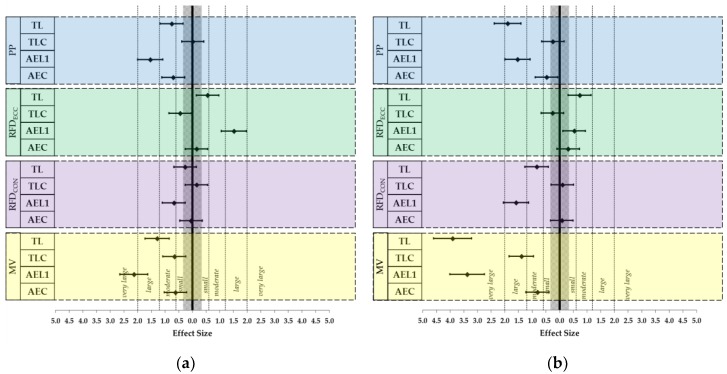
Within-condition Cohen’s *d* effect sizes ± 90% confidence interval for (**a**) the magnitude of change from repetition one to repetition three (∆REP_1–3_) and (**b**) the magnitude of change from repetition one to repetition five (∆REP_1–5_).

Descriptive statistics for each dependent variable are displayed in Table 1, Table 2, Table 3 and Table 4. Relative reliability of all dependent variables returned at least very large ICC (2,1) values, while absolute reliability of the dependent variables returned CV values ranging between 1.49–40.94% when considering all repetitions collected [13]. Within- and between-condition ES are presented in Figure 1 and Table 5, respectively. Concentric outputs tended to decrease in both straight-set configurations (TL and AEL1): peak power (*d* = −1.90 to −0.76), RFD_CON_ (*d* = −1.59 to −0.27), and MV (*d* = −3.91 to −1.29). Additionally, moderate decreases were observed for MV during both cluster conditions (*d* = −0.81 to −0.62). 

Accentuated eccentric clusters elicited greater RFD_ECC_ magnitudes in R3 and R5 compared to all other load conditions (*d* = 0.21–0.65). Conversely, small-to-moderate effect sizes indicated RFD_CON_ was greater during TLC than all other load conditions at R3 and R5 (*d* = 0.33–0.64). Consistent with concentric RFD, MV was greatest in the TLC condition. Relative to straight-set configurations (TL and AEL1), between-condition effect magnitudes became larger throughout the set, at R1 (*d* = 0.27–0.31, small), R3 (*d* = 0.67–0.72, moderate), and R5 (*d* = 1.34–1.51, large). Interestingly, the effect magnitudes between both cluster configurations (TLC and AEC) remained similar throughout the set, slightly favoring TLC (*d* = 0.30–0.42, small). Small-to-moderate effects indicated greater PP (*d* = 0.52) and MV (*d* = 0.61) during TLC compared to TL. However, only trivial effects were observed between TLC and AEC considering PP and MV changes.

## 4. Discussion

The purpose of this investigation was to explore the repetition-to-repetition kinetic and kinematic differences between potential programming tactics in the back squat. Specifically, the authors aimed to determine the effects of (1) eccentric overload and (2) inter-repetition rest on the magnitude and repetition-to-repetition changes of rate-related eccentric and concentric characteristics. In agreement with previous literature [32], the results of the current investigation suggest that the use of inter-repetition rest elicits a higher magnitude of peak power between conditions, paired with an increased ability to maintain peak power within a set compared to all load conditions through the initial three repetitions. This influence appears to be mainly driven by kinematic factors (i.e. MV). Accentuated eccentric loading does not appear to provide a potentiating effect on concentric output in straight-set or cluster-set configurations but may impart higher magnitude RFD_ECC_ compared to traditional loading.

Cluster sets have demonstrated efficacy as a method of inducing velocity and power adaptations [33,34]. Following a training program that included squats and weightlifting derivatives, Hansen and colleagues [34] demonstrated that the use of cluster sets throughout training caused greater changes in PP and peak velocity characteristics of a jump squat compared to the use of straight sets. Such chronic responses are likely related to the acute characteristics of cluster sets with higher velocity magnitudes within a session [35] and power output magnitudes within a set [36] observed using cluster set compared to straight set configurations. In agreement with previous literature, TLC resulted in greater concentric PP, RFD_CON_, and MV compared to straight set load conditions at R3 and R5. Interestingly, TLC also produced higher MV at R1 compared to all experimental conditions, potentially indicating that using TLC allows the carryover of less fatigue from set-to-set. Although, this may be the result of longer total rest compared to straight sets. Alternatively, this may indicate that intent is influenced by an athlete knowing whether an inter-repetition rest will be provided. Rationale aside, TLC permits the athlete an opportunity to express greater concentric outputs potentially advantageous in the later stages of a periodized training plan where such an emphasis is typically prescribed [37]. Moreover, ∆REP_1–3_ and ∆REP_1–5_ decreases were the least substantial in cluster configurations (TLC and AEC), further emphasizing its utility in maintaining concentric outputs across a set. This agrees with previous literature [32] and supports the efficacy of inter-repetition rest in acute management of fatigue. The application of eccentric overload during a cluster set (i.e. AEC) at least of the magnitude used in the current study caused a unique response. Higher magnitude MV were observed at R1, R3, and R5 using TLC compared to AEC. However, the ∆REP_1–5_ effect magnitude was less negative during AEC, indicating once again that intent may be influenced by the details of the loading strategy. The results comparing TLC and AEC suggest that the athletes may have been adjusting concentric intent to ensure sufficient energy was available to undertake the eccentric overload. Therefore, TLC may be most advantageous compared to AEC in maximizing the magnitude of concentric output, but AEC may be applied if maintenance within a set is desired.

A typical and theoretically-sound rationale for prescribing AEL in resistance training is to acutely potentiate the concentric output and has demonstrated effectiveness in the previous literature using bench press and squats [8,12,23]. However, evidence that AEL does not elicit a potentiating response is similarly prevalent [38] though the relative inconsistency in loading means and magnitude makes drawing definitive conclusions problematic. The current investigation is the first to consider repetition-to-repetition magnitudes and within-set changes using two different AEL strategies, though these strategies have been explored from the training session-level in prior study [13]. As previously discussed, considering R1 before significant accumulation of fatigue would theoretically be experienced and immediately preceded by full recovery, the application of eccentric overload induced small detrimental effects on MV magnitude compared to TLC. Interestingly, RFD_CON_ was greater at R1 when eccentric overload was prescribed during straight sets, but lower when applied to a cluster set. Though initially appearing to add to the convoluted nature of the evidence regarding the potentiating effects of AEL, the between-condition effects on RFD_CON_ and MV worsened at R3 and R5 compared to traditionally loaded conditions, suggesting a fatiguing effect from AEL. Providing further support, within-condition ∆REP_1–3_ decreases in RFD_CON_ and MV were also larger when eccentric overload was applied to straight sets. However, because ∆REP_1–3_ and ∆REP_1–5_ were similar between TLC and AEC, changes in intent should again be considered as a rationale. 

Though the current investigation presented evidence supporting the potentially fatiguing nature of AEL, this may be due to a sensitivity in concentric or eccentric load prescription rather than a generalizable conclusion regarding eccentric overload. More important may be the presence of kinetic characteristics that have demonstrated efficacy in potentiating concentric outputs. For example, when high RFD_ECC_ is present, it is possible that a greater muscle spindle activation [39] or a pre-attachment of cross-bridges via Ca^2+^ influx [40] occur both of which contribute to acute concentric potentiation so long as the eccentric and concentric action are tightly coupled [26]. Higher magnitude RFD_ECC_ was observed in AEC compared to TLC, providing a mechanistic rationale for induction of acute potentiation via AEL. Further, a large within-condition ∆REP_1–3_ for RFD_ECC_ was present using AEL1. This suggests that despite overload being applied during R1 only, the enhancement in RFD_ECC_ may continue for at least three repetitions. The effect at ∆REP_1–5_ reduced to small and a lower magnitude RFD_ECC_ was produced at R5 compared to R3, meaning that if this eccentric facilitation were desired, three repetitions within a set may be more optimal. This provides important practical considerations for coaches, as weight releasers may not need to be reapplied at each repetition to enhance RFD_ECC_ within a set. Despite convincing evidence that RFD_ECC_ is enhanced using AEL, this did not correspond with the expected comparatively higher concentric outputs (i.e. PP, RFD_CON_, MV). It is possible then, that the eccentric overload prescription produced the desired outcome, but the concentric load prescription may need to be lowered to produce acute concentric potentiation. Previous investigations have explored the effects of different magnitudes of eccentric overload on potentiation at a fixed concentric load [8,12]. However, future investigations should consider the opposite: how manipulating the concentric prescription accompanied by a fixed eccentric overload influences acute potentiation. 

## 5. Conclusions

The results of the current investigation demonstrate that inter-repetition rest permits higher magnitude and improved maintenance of kinetic and kinematic concentric outputs throughout a set. Further, AEL does not appear to provide a potentiating effect on concentric output in straight-set or cluster-set configurations but may impart higher magnitude RFD_ECC_ compared to traditional loading therefore providing the mechanistic characteristics to theoretically potentiate concentric outputs. Though potentiation was not observed in the current investigation, future study should focus on different concentric and eccentric load prescriptions using AEL to determine if concentric potentiation is prescription, rather than method-sensitive, in the back squat. Finally, important practical considerations were elucidated in applying eccentric overload for the initial repetition of the set. The results of the current investigation suggest that applying eccentric overload for the initial repetition of a set only may alter RFD_ECC_ substantially for at least two subsequent traditionally loaded repetitions. There were limitations to the current investigation that may have influenced the outcomes including differences in work and work-to-rest ratios between load conditions. However, this was a purposeful aspect of the design in order to make it a more practical comparison. 

## Figures and Tables

**Table 1:** Concentric peak power presented as mean (M) ± 90% confidence interval (CI).

Repetition	PP (W)											
	TL			TLC			AEL1			AEC		
R1	2638.12	±	241.45	2869.44	±	300.62	2704.62	±	272.97	2797.67	±	295.10
R3	2496.74	±	221.79	2844.20	±	282.82	2525.61	±	244.91	2627.10	±	228.15
R5	2364.68	±	203.80	2791.61	±	276.63	2415.14	±	228.50	2651.61	±	212.77
∆REP_1–3_	−141.38	±	52.67	−25.24	±	31.98	−179.01	±	56.18	−170.57	±	169.53
∆REP_1–5_	−273.44	±	83.10	−77.83	±	56.94	−289.48	±	60.62	−146.06	±	151.38

PP = peak power; R1 = first repetition; R3 = third repetition; R5 = fifth repetition; ∆REP_1–3_ = change from first repetition to third repetition; ∆REP_1–5_ = change from first repetition to fifth repetition.

**Table 2:** Eccentric rate of force development presented as mean (M) ± 90% confidence interval (CI).

Repetition	RFD_ECC_ (N/s)											
	TL			TLC			AEL1			AEC		
R1	2515.93	±	329.17	2752.57	±	336.82	2766.49	±	528.00	3115.18	±	372.94
R3	2735.06	±	373.72	2412.35	±	316.22	2943.66	±	403.30	3237.90	±	409.44
R5	2764.42	±	358.83	2448.90	±	324.01	2816.68	±	375.33	3270.97	±	461.88
∆REP_1–3_	219.13	±	170.26	−340.21	±	235.77	177.17	±	660.08	122.72	±	314.70
∆REP_1–5_	248.49	±	103.48	−303.67	±	227.92	50.19	±	684.15	155.80	±	414.89

RFD_ECC_ = eccentric rate of force development; R1 = first repetition; R3 = third repetition; R5 = fifth repetition; ∆REP_1–3_ = change from first repetition to third repetition; ∆REP_1–5_ = change from first repetition to fifth repetition.

**Table 3:** Concentric rate of force development presented as mean (M) ± 90% confidence interval (CI).

Repetition	RFD_CON_ (N/s)											
	TL			TLC			AEL1			AEC		
R1	1518.94	±	223.43	1863.61	±	260.99	1704.26	±	311.61	1629.89	±	289.27
R3	1440.05	±	234.43	1906.43	±	297.33	1401.40	±	230.31	1583.12	±	265.56
R5	1386.14	±	260.16	1901.80	±	306.73	1318.00	±	206.97	1542.21	±	255.12
∆REP_1–3_	−78.90	±	61.15	42.82	±	81.31	−302.86	±	114.53	−46.77	±	179.36
∆REP_1–5_	−174.81	±	75.17	38.19	±	89.11	−386.27	±	128.38	−87.68	±	199.46

RFD_CON_ = concentric rate of force development; R1 = first repetition; R3 = third repetition; R5 = fifth repetition; ∆REP_1–3_ = change from first repetition to third repetition; ∆REP_1–5_ = change from first repetition to fifth repetition.

**Table 4:** Concentric average velocity presented as mean (M) ± 90% confidence interval (CI).

Repetition	MV (m/s)											
	TL			TLC			AEL1			AEC		
R1	0.54	±	0.02	0.56	±	0.02	0.54	±	0.02	0.54	±	0.02
R3	0.49	±	0.02	0.54	±	0.02	0.48	±	0.03	0.51	±	0.02
R5	0.43	±	0.02	0.52	±	0.02	0.42	±	0.02	0.49	±	0.02
∆REP_1–3_	−0.05	±	0.01	−0.02	±	0.00	−0.06	±	0.01	−0.03	±	0.02
∆REP_1–5_	−0.11	±	0.01	−0.04	±	0.01	−0.12	±	0.01	−0.05	±	0.02

MV = average concentric velocity; R1 = first repetition; R3 = third repetition; R5 = fifth repetition; ∆REP_1–3_ = change from first repetition to third repetition; ∆REP_1–5_ = change from first repetition to fifth repetition.

**Table 5:** Between-condition Cohen's *d* effect sizes ± 90% confidence interval.

Repetition	PP					RFD_ECC_			RFD_CON_			MV		
R1	AEL1	TL	0.09	±	0.41	0.16	±	0.41	0.20	±	0.41	0.02	±	0.40
		TLC	−0.16	±	0.41	0.01	±	0.40	−0.16	±	0.41	−0.27	±	0.41
		AEC	−0.09	±	0.41	−0.22	±	0.41	0.07	±	0.41	0.03	±	0.40
	TLC	TL	0.30	±	0.41	0.20	±	0.41	0.41	±	0.41	0.31	±	0.41
		AEC	0.07	±	0.41	−0.29	±	0.41	0.24	±	0.41	0.30	±	0.41
	AEC	TL	0.21	±	0.41	0.49	±	0.41	0.12	±	0.41	−0.01	±	0.40
R3	AEL1	TL	0.04	±	0.41	0.15	±	0.41	−0.05	±	0.41	−0.18	±	0.41
		TLC	−0.34	±	0.41	0.42	±	0.41	−0.54	±	0.41	−0.72	±	0.42
		AEC	−0.12	±	0.41	−0.21	±	0.41	−0.21	±	0.41	−0.34	±	0.41
	TLC	TL	0.39	±	0.41	−0.27	±	0.41	0.50	±	0.41	0.67	±	0.42
		AEC	0.24	±	0.41	−0.65	±	0.42	0.33	±	0.41	0.42	±	0.41
	AEC	TL	0.17	±	0.41	0.37	±	0.41	0.16	±	0.41	0.21	±	0.41
R5	AEL1	TL	0.07	±	0.41	0.04	±	0.41	−0.08	±	0.41	−0.06	±	0.41
		TLC	−0.42	±	0.41	0.30	±	0.41	−0.64	±	0.42	−1.34	±	0.45
		AEC	−0.31	±	0.41	−0.31	±	0.41	−0.28	±	0.41	−0.88	±	0.42
	TLC	TL	0.50	±	0.41	−0.26	±	0.41	0.52	±	0.41	1.51	±	0.46
		AEC	0.16	±	0.41	−0.59	±	0.41	0.36	±	0.41	0.40	±	0.41
	AEC	TL	0.39	±	0.41	0.35	±	0.41	0.17	±	0.41	0.95	±	0.43

PP = peak power; RFD_ECC_ = eccentric rate of force development; RFD_CON_ = concentric rate of force development; MV = average concentric velocity; R1 = first repetition; R3 = third repetition; R5 = fifth repetition.
